# Detecting DNA synthesis of neointimal formation after catheter balloon injury in GK and in Wistar rats: using 5-ethynyl-2'-deoxyuridine

**DOI:** 10.1186/1475-2840-11-150

**Published:** 2012-12-13

**Authors:** Jingsheng Guo, Dongye Li, Shiru Bai, Tongda Xu, Zhongmin Zhou, Yanbin Zhang

**Affiliations:** 1 Institute of Cardiovascular Disease Research, Xuzhou Medical College, 84 West Huaihai Road, Xuzhou, Jiangsu, Peoples Republic of China; 2 Department of Internal Medicine, Aultman Hospital & Canton Medical Education Foundation, Northeast Ohio Medical University, Canton, OH, USA

**Keywords:** Neointimal formation, DNA synthesis, Diabetes mellitus, EdU, Catheter balloon injury, PCNA

## Abstract

**Background:**

Neointimal formation plays an important role in the pathogenesis of coronary restenosis after percutaneous coronary intervention (PCI), especially in patients with diabetes mellitus. Recently, some studies have shown that 5-ethynyl-2'-deoxyuridine (EdU) incorporation can serve as a novel alternative to the 5-bromo-2'-deoxyuridine (BrdU) antibody detection method for detection of DNA synthesis in regenerating avian cochlea, chick embryo and the adult nervous system. However, few studies have been performed to assess the suitability of EdU for detecting DNA synthesis in vascular neointima.

**Methods:**

The carotid artery balloon injury model was established in Goto-Kakizaki (GK) and Wistar rats. A Cell-Light^TM^ EdU Kit was used to detect EdU-labeled cell nuclei of common carotid arteries at day 7 after catheter balloon injury. Different methods of injecting EdU were tested. The protein levels of proliferating cell nuclear antigen (PCNA) and p-Akt (Ser473), as well as the mRNA levels of PCNA were evaluated by Western blotting and quantitative real-time PCR (qRT-PCR), respectively. Immunohistochemical staining was also employed to visualize PCNA-positive cells.

**Results:**

At day 7 after catheter balloon injury, far more EdU-positive and PCNA-positive cells were observed in GK rats. When comparing groups that received different EdU doses, it was found that the percentage of EdU-positive cells at a dose of 100 mg/kg body weight was than at doses of 25 mg/kg and 50 mg/kg. The number of positive cells was significantly higher in the repeated injection group compared to the single injection group. Further, after balloon injury DNA synthesis in GK rats was more notable than in Wistar rats. Neointimal formation in GK rats was more obvious than in Wistar rats. The protein levels of PCNA and p-Akt (Ser473) and the mRNA levels of PCNA were increased in injured rats as compared to uninjured rats, and were significantly higher in GK rats than in Wistar rats.

**Conclusion:**

By intraperitoneal injections of EdU at a dose of 100 mg/kg three times, EdU incorporation can detect carotid arterial DNA synthesis caused by neointimal formation in GK rats and Wistar rats at day 7 after balloon injury by the EdU click reaction quickly and effectively. Moreover, more obvious DNA synthesis in the vascular neointima could be observed in GK rats than in Wistar rats.

## Background

Coronary artery disease (CAD) is the leading cause of death among both men and women in China and western industrialized nations. Diabetes mellitus is the single worst risk factor for CAD. CAD in diabetic patients is diffuse, severe, presents with more complex lesions and often progresses rapidly. Restenosis remains the major limitation for long-term success after percutaneous coronary intervention (PCI) even in the drug eluting stent era, especially in patients with diabetes mellitus [1,2]. Angiographic restenosis rates of 15.2% have been demonstrated following successful drug-eluting stenting among patients with diabetes in a study in Germany [3]. Drug eluting stents are considered by many to be the standard of care for patients with diabetes undergoing PCI; however, analysis of diabetic subgroup in the SIRIUS (SIRolImUS-coated Bx Velocity balloon-expandable stent in the treatment of patients with de novo coronary artery lesions) trial demonstrated that absolute late loss of lumen and restenosis remains higher in patients with diabetes receiving stents eluting sirolimus (also known as rapamycin, an immunosuppressive agent) [4]. TAXUS (a brand of stent) trials showed similar results, in which the slow rate-release polymer-based stents eluting paclitaxel (an antineoplastic agent) were employed [5]. Sheehy *et al.* found that the use of paclitaxel-eluting stents resulted in greater neointimal area and increased inflammation compared to everolimus-eluting stents [6]. Thus, it is clear that the occurrence of restenosis still remains inevitable regardless which drug-eluting stent is employed. Generally, restenosis is the result of early elastic recoil, adverse remodeling, and the formation of neointimal after angioplasty or stenting. Additionally, vascular smooth muscle cell (VSMC) proliferation is one of major mechanisms of neointimal formation. Some studies have been conducted to investigate the molecular mechanism by which it occurs [7,8] and have tried to suppress neointimal formation by attenuating VSMC proliferation [9]. Therefore, observation of VSMCs proliferation and neointimal formation is an important method for studying restenosis after vascular injury. Detecting DNA synthesis in the vascular wall can indirectly reflect the VSMC proliferation *in vivo*.

Five-ethynyl-2'-deoxyuridine (EdU) is a nucleoside analog of thymidine which can be incorporated into DNA during active DNA synthesis. It is considered to be a novel alternative for 5-bromo-2'-deoxyuridine (BrdU) to measure DNA synthesis or S-phase synthesis of the cell cycle [10,11]. BrdU staining requires harsh DNA denaturation (HCl, digestion with DNase or heat) to expose BrdU for detection with an anti-BrdU antibody and this action can destroy the tissue’s structures. In marked contrast, detection of EdU is based on a copper (I) catalyzed reaction [12,13] between an azide and an alkyne, and this reaction is considerably faster. Using an azide-containing detection reagent to react with the alkyne contained in EdU can form a stable triazole ring. Moreover, the azide-containing detection reagent is significantly smaller than a BrdU antibody, so it can penetrate into a double-stranded DNA easily and can react with incorporated EdU without denaturation of the DNA. The advantage of a “click” reaction is that it makes it more convenient to observe DNA synthesis *in vitro*[14-17] and allows for a simple, fast protocol which produces more reproducible and reliable results. The “click” reaction involves the coupling of an azide-labeled compound and a terminal alkyne-labeled compound in the presence of copper (I) to form a stable covalent triazole ring conjugate [11]. At the same time, some recent studies have shown that EdU can also be used to observe DNA synthesis *in vivo*[18-21].

A common factor in the pathogenesis restenosis after PTCA (Percutaneous transluminal coronary angioplasty) and PCI is neointimal formation, which appears following catheter balloon injury. In our previous studies, we used the carotid artery balloon injury model in obese Zucker rats, a well-established model of type II diabetes mellitus (T2DM) [22], as well as lean Zucker rats, to investigate the vascular response to balloon injury in diabetes [23-25]. Our studies demonstrated that this model is still valuable for investigating the mechanism of neointimal formation. Moreover, we have observed neointimal formation using the BrdU antibody detection method [24,26]. We found that the number of BrdU-positive cells in obese Zucker rats after balloon injury was more apparent than in lean Zucker rats. However, few studies have been done on whether EdU can be used to detect DNA synthesis of neointimal formation. The Zucker obese rat and Goto-kakizaki (GK) rat are both established rat models of T2DM. The GK rat model was developed by selectively breeding non-diabetic Wistar rats for glucose intolerance over multiple generations [27]. Compared with Zucker obese rats, GK rats are also characterized by hyperinsulinemia and mild hyperglycemia but are not obese, and have a stable and inheritable form of T2DM [28]. And the use of GK rats is more cost-effective due to the fact that their traits are dominant. The aim of the present study is to examine whether EdU incorporation can detect DNA synthesis in the vascular neointima of GK rats and Wistar rats after balloon injury by the EdU click reaction quickly and effectively.

## Methods

### Animals

Male GK rats and male Wistar rats (weight, average 380 g) were used as a diabetic group and a non-diabetic group, respectively. Animals were divided into four subgroups: GK injury, GK control, Wistar injury and Wistar control. Nine rats were selected in each group. All animals and forage were purchased from SLAC Laboratory Animal Limited Company, Shanghai. GK rats were fed with a high fat diet and Wistar rats with a normal diet. Drink and food were given randomly. The room temperature was maintained at 23 to 25 degrees Celsius,with 50%-60% humidity and eight hours of light per day. All experiments were approved by the Animal Research Committee of Xuzhou Medical College (permit number XMCACUC2010-08-114).

### Carotid artery balloon injury model in rats

All rats were anesthetized by intraperitoneal injection of 10% chloral hydrate (3 ml/kg body wt) diluted in double distilled water. Surgical procedures were performed by sterile surgical technique. A midline cervical incision was made to expose the left external carotid artery by blunt dissection. A 2F Fogarty balloon catheter (Edwards Lifescience Corp) was introduced from the external carotid artery into the aortic arch after the distal end of the external carotid artery was ligated. The balloon was distended with saline until a slight resistance was felt on slight traction. The balloon was gently rotated and withdrawn into the common carotid artery, then pulled back into the external carotid artery. After this procedure was repeated 3 times, the proximal end was ligated to the external carotid artery. At day 7 after balloon injury, the left common carotid arteries were completely exposed and removed after rats were euthanized by intraperitoneal injection with 10% chloral hydrate. Harvested arteries were flushed rapidly with ice-cold phosphate buffer saline (PBS). These specimens were stored in 4% paraformaldehyde diluted in PBS for 24 to 48 hours before paraffin embedding or frozen in negative 86 degrees freezer (Thermo Scientific Forma ULT).

### EdU staining

EdU staining was conducted using Cell-Light^TM^ EdU Kit (Rui Bo Guangzhou Biotechnology Limited Company, China), according to the manufacturer’s protocol. Paraffin sections were de-paraffinated in xylene for 10 min twice, a gradient of alcohol (100%, 95%, 85%) for 10 min, and were rinsed in deionized water for 5 min. After washing with 2 mg/ml glycine solution diluted in double distilled water for 10 min, the sections were permeablized with 0.5% Triton X-100 in PBS for 20 min, and then washed twice with PBS for 10 min each rinse. The Apollo reaction buffer liquid, catalyst, fluorescent dyes and buffer additives were dissolved in deionized water, and shaken to make the Apollo® 567 staining reaction solution. The sections were then incubated for 30 min without light. The sections were washed twice with PBS for 10 min each rinse. For subsequent DNA staining, sections were counterstained with Hoechst 33342 for 30 min away from light. The slides were then washed twice with PBS for 3 min each rinse, and observed immediately under a fluorescent microscope (magnification ×400). All the procedures were conducted at room temperature. An Olympus BX51 microscope (Olympus, Japan) was used to observe EdU-positive cells. Images of the Apollo® 567 were captured with a “red” filter set: Excitation: 550nm, Emission: 565nm, Filter: 555±15nm. Images of the Hoechst 33342 were captured with a “blue” filter set: Excitation: 350nm, Emission: 461nm, Filter: 405±15nm.

### Quantitative real-time PCR (qRT-PCR)

Total RNA was isolated from common carotid artery of Wistar and GK injury and control, using TRIzol® Reagent (Invitrogen life technologies) according to the manufacturer's instructions. Two micrograms of total RNA was then used for reverse transcription reaction using MMLV reverse transcriptase (Epicentre, Madison, WI). qRT-PCR was performed in an ABI PRISM7900 system (Applied Biosystems, Foster City, CA). And the gene expression of proliferating cell nuclear antigens (PCNA) and GAPDH was examined by 2×PCR master mix (Superarray). The reaction protocol included preincubation at 95°C to activate Taq DNA polymerase for 10 min, amplification of 40 cycles that was set for 10 s at 95°C, and annealing for 60 s at 60°C. The results were normalized with the housekeeping gene rat GADPH. Primer sequences were designed using software Primer 5.0 as follows. Rat PCNA forward, 5’TGAAGTTTTCTGCGAGTGGG3’; rat PCNA reverse, 5’CAGTGGAGTGGCTTTTGTGAA3’; rat GADPH forward, 5’GGAAAGCTGTGGCGTGAT-3’; rat GADPH reverse, 5’AAGGTGGAAGAATGGGAGTT3’.

### Western blot analysis

The frozen common carotid tissue was homogenized in 100ul of ice-cold RIPA Lysis Buffer including 1μL PMSF (100 mM) (Beyotime Institute of Biotechnology, China) and then incubated on ice for 30 min. Samples were centrifuged at 12,000g for 10 min at 4°C. The supernatant was collected and measured for total protein content using the BCA Protein Assay kit (Beyotime Institute of Biotechnology, China). 20 μg total lysate was run on SDS-polyacrylamide gel electrophoresis and transferred onto Polyvinylidene Fluoride (PVDF) membranes. Membranes were blocked in TBST buffer containing 5% Difco TM Skim Milk (BD 232100) and incubated with the primary antibodies PCNA, Akt and p-Akt (Ser 473) (Cell Signaling Technology). Secondary antibodies were horseradish peroxidase (HRP) labeled Goat Anti-Mouse IgG (H+L), and detection was performed using enhanced chemiluminescence (ECL) (Beyotime Institute of Biotechnology, China). Measurements of band density were performed using Image J for Windows software (National Institutes of Health). Each experiment was repeated three times.

### Immunohistochemistry for PCNA

Immunohistochemistry was performed using mouse monoclonal antibody to (Cell Signaling Technology) PCNA as the first antibody. SP (Streptavidin-Peroxidase) immunohistochemical assay Kit (Zhongshan Goldenbridge Biotechnology CO. LTD) was used during the staining procedure according to the manufacturer’s protocol.

### Morphometry and histopathology

The arterial segments were embedded in paraffin. Sections (3~5 μm thick) were de-paraffinated and underwent hematoxylin-eosin (H&E) staining and Elastic Van Gieson (E.V.G) staining. Histomorphometric analysis was performed by individuals blinded to the treatment mode. The ratio of neointimal to medial area (N/M) was calculated using Image Pro Plus 3.0 (Media Cybernetics Corporation)

### Blood glucose and lipid levels

At the time of euthanasia, blood samples were collected by cardiac puncture. Serum glucose levels, total cholesterol, HDL cholesterol, LDL cholesterol and triglycerides were measured by the enzymatic method analysis.

### Statistical analysis

Quantitative data were expressed as mean±SD. Statistical analyses was performed with the use of GraphPad Prism 5.0 (GraphPad Software Inc). Continuous variables were compared using student t tests. A value of p<0.05 was considered to be statistically significant.

## Results

### EdU staining for DNA synthesis

**Figure 1:**
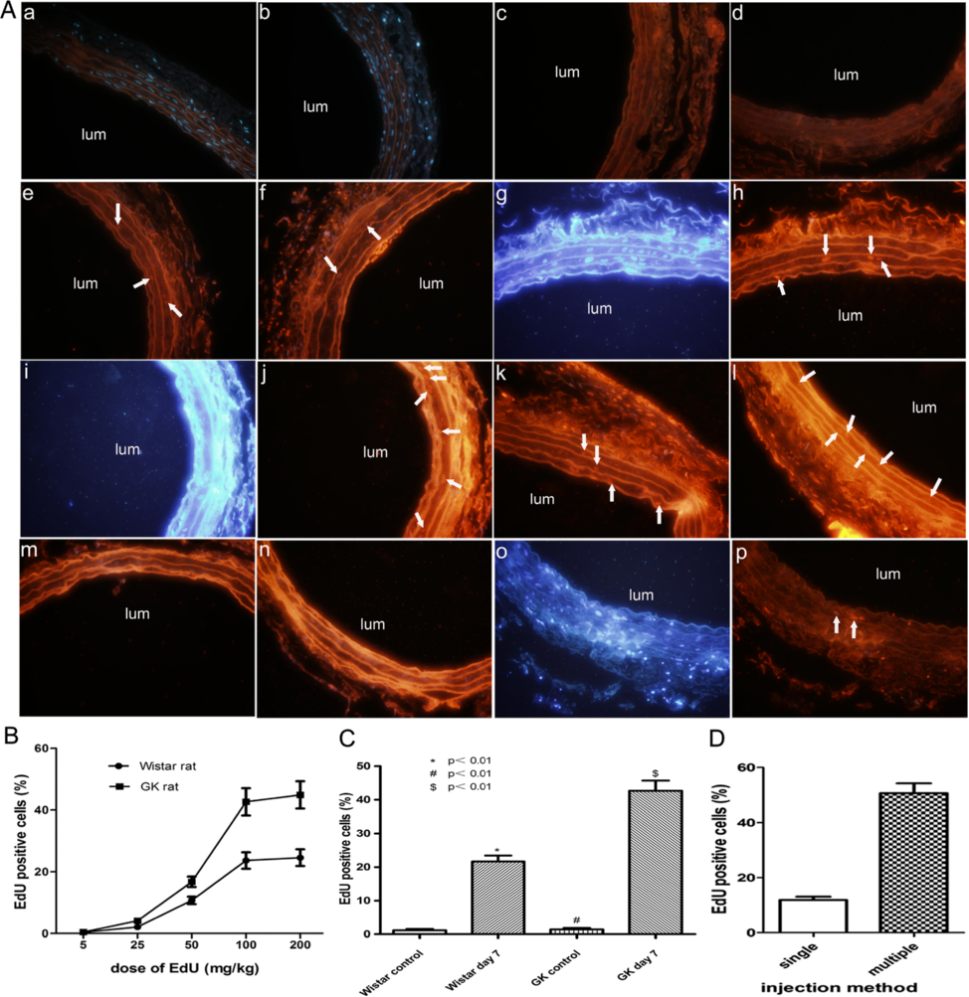
**Representative images of EdU staining by different EdU injection methods (magnification ×400).** (**A**) Three intraperitoneal injections of EdU at day 7 after balloon injury (**a**-**l**): (**a**, **c**, **e**) EdU staining for Wistar rats at a dose of 5 mg/kg, 25 mg/kg and 50 mg/kg in sequence; (**b**, **d**, **f**) EdU staining for GK rats at a dose of 5 mg/kg, 25 mg/kg and 50 mg/kg in sequence; (**g**-**j**) Hoechst 33342 and EdU staining for Wistar and GK rats at a dose of 100 mg/kg in order; (**k**, **l**) EdU staining for Wistar and GK rats at a dose of 200 mg/kg, respectively. (m, n) EdU staining for uninjuried Wistar and GK rats at a dose of 100 mg/kg, respectively. (**o**, **p**) Hoechst 33342 and EdU staining for GK rats at a single injection of 100 mg/kg, respectively. (**B**) Line charts depicting the dose dependence of EdU staining. (**C**) Bar diagrams showing the increased number of EdU-positive cells was more significant in the injury groups than in the uninjured groups for Wistar and GK rats at a dose of 100 mg/kg, respectively. (**D**) Bar diagrams showing a greater number of EdU-positive cells were observed using multiple injections than a single one. Arrows in images sign EdU-labeled cells and lums indicate luminal side of artery. * indicates Wistar control versus Wistar day 7, # indicates GK control versus GK day 7, $ indicates Wistar day 7 versus GK day 7. p<0.01.

The percentage of EdU-positive cells in GK and Wistar rats using different doses of EdU was studied. EdU dissolved in PBS was injected intraperitoneally into Wistar and GK rats using the following doses: 5, 25, 50, 100 and 200 mg/kg at hours 18, 12 and 2 before the euthanasia. Common carotid arteries were harvested for fixation and embedding. EdU staining was then conducted according to the manufacturer’s protocol. Few EdU-positive cells were observed in Wistar and GK rats at a dose of 5 mg/kg and 25 mg/kg (Figure 1A. a-d). A few EdU-positive cells were seen in Wistar and GK rats at a dose of 50 mg/kg (Figure 1A. e-f). Prominent EdU-positive cells were observed in Wistar and GK rats at a dose of 100 mg/kg and 200 mg/kg, respectively (Figure 1A. g-l). As shown in Figure 1B, the dose dependence of EdU staining was found and using 100 mg/kg met the basic saturated EdU staining result. And the increased number of positive cells were found in the injury group than in the control group at a dose of 100 mg/kg for both groups: GK rats (42.67±3.07% versus 1.42±0.42%, p<0.01) and Wistar rats (21.67±1.76% versus 1.15±0.38%, p<0.01); the percentage was higher in GK rats (p<0.01) (Figure 1A. g-j, m-n, and C).

Whether a single injection could achieve the same result was also studied. First, EdU was injected intraperitoneally (100 mg/kg) into injured GK rats at hours 4 before euthanasia. Second, EdU was injected intraperitoneally (100 mg/kg) three times at hours 18, 12 and 2 before the euthanasia. The common carotid arteries were harvested and EdU staining was conducted as described above. A greater number of EdU-positive cells were observed using multiple injections than a single one (p<0.01) (Figure 1A. i-j, o-p and D).

### qRT-PCR analysis of PCNA mRNA expression

**Figure 2:**
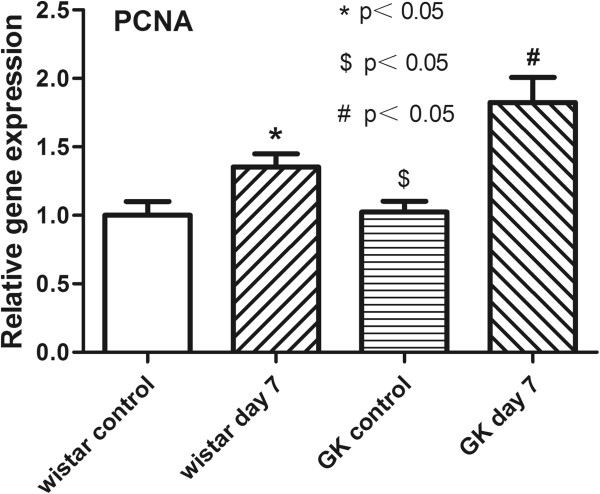
**qRT-PCR of PCNA from common carotid arteries in both GK and Wistar rats.** The fold change was calculated compared with the expression of Wistar control group (set as value 1). Data represent the mean±SD, and the experiment was repeated three times. * indicates Wistar control versus Wistar day 7, # indicates GK control versus GK day 7, $ indicates Wistar day 7 versus GK day 7. p<0.05.

In order to further validate the cell proliferation at the level of gene expression, balloon injury-induced gene transcription of PCNA was examined by qRT-PCR. As shown in Figure 2, PCNA expression was markedly up-regulated at day 7 after balloon injury in Wistar rats (1.35±0.05-fold versus 1.00±0.06-fold, p<0.05) and GK rats (1.82±0.11-fold versus 1.02±0.05-fold, p<0.05), and was significantly higher in injured GK arteries than in injured Wistar arteries (p<0.05). There was no significant difference found between the uninjured arteries of GK and Wistar rats (p>0.05).

### PCNA and p-Akt protein expression levels

**Figure 3:**
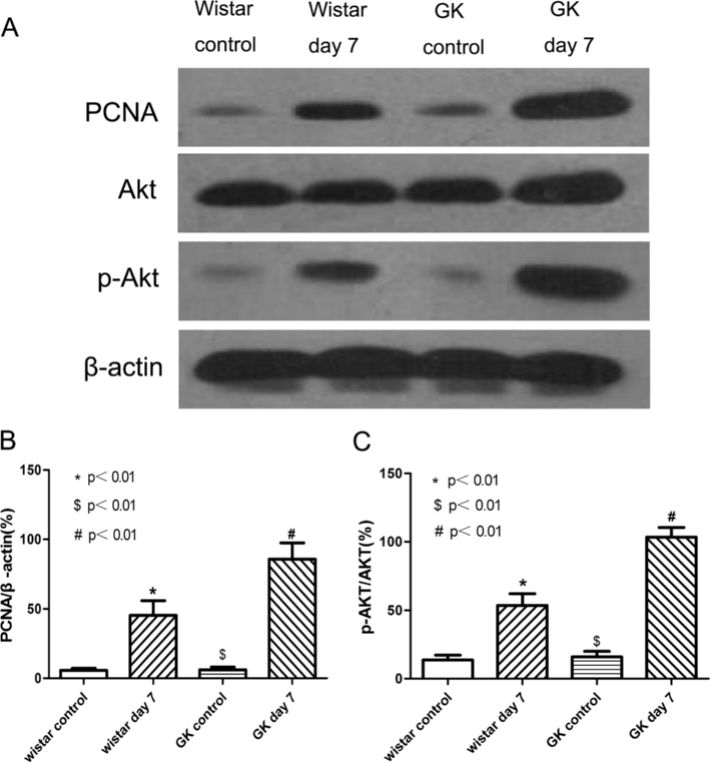
**Balloon injury-induced activation of PCNA and p-Akt in common carotid arteries.** (**A**) Representative Western blot for PCNA, Akt and p-Akt in GK and Wistar rats at day 7 after balloon injury and control groups. (**B**) Bar diagrams depicting the relative protein level of PCNA after normalization to β-actin. (**C**) Bar diagrams showing the ratio of p-Akt to Akt. Data represent the mean±SD, n=3. * indicates Wistar control versus Wistar day 7, # indicates GK control versus GK day 7, $ indicates Wistar day 7 versus GK day 7. p<0.01.

PCNA and p-Akt levels were measured in Wistar and GK rat arteries after injury and in controls by Western blotting. The results, shown in Figure 3, demonstrate a basal level of PCNA and p-Akt protein expression exists in uninjured rats, but an increase in expression occurs in response to endothelial injury. Western blot analysis revealed that PCNA expression increased both in GK rats (85.67±6.74% versus 6.00±1.16%, p<0.01) and in Wistar rats (45.33±6.12% versus 5.67±0.88%, p<0.01) after balloon injury, and the increase in the former was more significant than the latter (p<0.01). The phosphorylation level of Akt, which was expressed at the ratio of p-Akt to Akt, which was significantly higher in GK rats after balloon injury (99.67±3.28% versus 16.00±2.31%, p<0.01) than was observed in Wistar rats (53.67±4.91% versus 13.67±2.03%, p<0.01). There was no significant difference found between the GK and Wistar control groups in the levels of either PCNA or p-Akt/Akt (p>0.05).

### PCNA Immunohistochemical analysis

**Figure 4:**
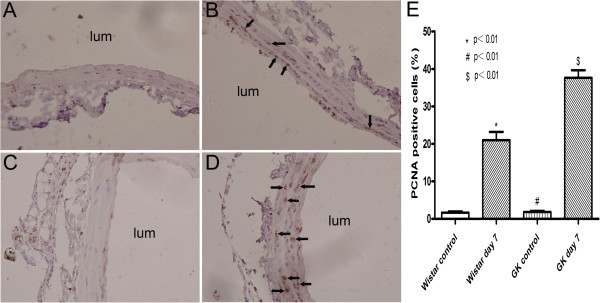
**Representative immunohistochemical photomicrographs for PCNA expression in common carotid arteries (magnification ×400).** (**A**) Wistar rats control. (**B**) A large number of PCNA positive cells were observed in Wistar rats at day 7 after balloon injury. (**C**) GK rats control. (**D**) Strong PCNA immunostaining was seen in GK rats at day 7 after balloon injury. (**E**) The percentage of PCNA positive cells in Wistar and GK rats after balloon injury increased significantly as compared to Wistar and GK rats control respectively. The percentage of PCNA positive cells in Wistar rats was significantly less than in GK rats. Arrows in images sign PCNA positive cells and lums indicate luminal side of artery. * indicates Wistar control versus Wistar day 7, # indicates GK control versus GK day 7, $ indicates Wistar day 7 versus GK day 7. p<0.01.

PCNA monoclonal antibody was used for immunohistochemical staining in GK and Wistar rats before and after carotid artery injury for both groups. PCNA-positive cells increased significantly in common carotid arteries at day 7 after balloon injury than before the injury in GK (37.67±1.99% versus 1.83±0.31%, p<0.01) and in Wistar rats (21.00±2.21% versus 1.67±0.33%, p<0.01). PCNA positive cells in GK rats were more than those in Wistar rats (p<0.01) (Figure 4).

### Morphology and histopathology analysis

**Figure 5:**
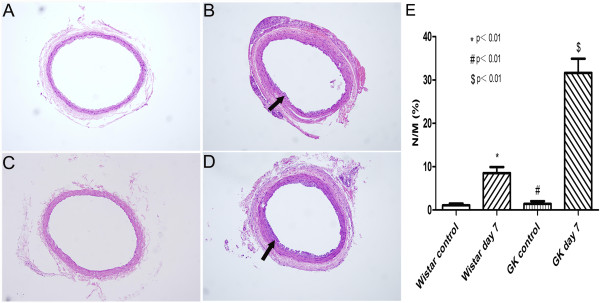
**Morphological changes of rat common carotid arteries by H&E staining method (magnification ×200).** (**A**) Wistar rats control. (**B**) Neointimal formation was seen in Wistar rats at day 7 after balloon injury. (**C**) GK rats control. (**D**) Visible neointimal formation was observed in GK rats at day 7 after balloon injury. (**E**) The degree of neointimal hyperplasia expressed by N/M in Wistar and GK rats after balloon injury increased significantly as compared to Wistar and GK rats control, respectively. N/M in GK rats was significantly higher than in Wistar rats. Arrows in images sign neointimal formation after balloon injury. * indicates Wistar control versus Wistar day 7, # indicates GK control versus GK day 7, $ indicates Wistar day 7 versus GK day 7. p<0.01.

**Figure 6:**
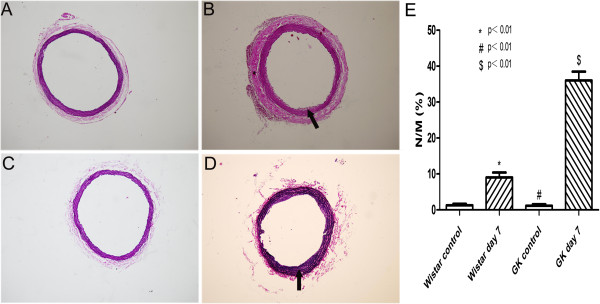
**Morphological changes of rat common carotid arteries by E.V.G staining method (magnification ×200).** (**A**) Wistar rats control. (**B**) Neointimal formation was seen in Wistar rats at day 7 after balloon injury. (**C**) GK rats control. (**D**) Visible neointimal formation was observed in GK rats at day 7 after balloon injury. (**E**) The degree of neointimal hyperplasia expressed by N/M in Wistar and GK rats after balloon injury increased significantly as compared to Wistar and GK rats control, respectively. N/M in GK rats was significantly higher than in Wistar rats. Arrows in images sign neointimal formation after balloon injury. * indicates Wistar control versus Wistar day 7, # indicates GK control versus GK day 7, $ indicates Wistar day 7 versus GK day 7. p<0.01.

The common carotid arteries were harvested for H&E and E.V.G staining at day 7 after balloon injury. More visible neointimal formation could be observed in common carotid arteries of GK rats after balloon injury than before the injury (36.00±2.48% versus 1.15±0.41%, p<0.01). The same result can also be observed in injured Wistar rats (9.00±1.42% versus 1.28±0.42, p<0.01). At the same time, neointimal formation after balloon injury in GK rats were compared to that in Wistar rats. More visible neointimal formation could be observed in injured GK rats than in injured Wistar rats (p<0.01) (Figure 5 and Figure 6).

### Blood biochemistry test

The level of serum glucose, total cholesterol, HDL cholesterol, LDL cholesterol and triglycerides in GK rats and Wistar rats was measured and is shown in Table 1.

**Table 1:** Serum glucose and lipids levels of GK and Wistar rats after injury

***Serum***	***GK rats(n=9)***	***Wistar rats(n=9)***	***p-value***
GLU (mmol/L)	18.96±0.74	12.01±0.58	<0.001
CHO (mmol/L)	2.81±0.20	1.23±0.06	<0.001
LDL-C (mmol/L)	1.59±0.05	0.65±0.05	<0.001
HDL-C (mmol/L)	0.95±0.05	0.45±0.03	<0.001
TG (mmol/L)	0.94±0.03	1.04±0.22	>0.05

GLU, serum glucose level; CHO, total cholesterol; LDL-C, low density lipoprotein cholesterol; HDL-C, high density lipoprotein cholesterol.

## Discussion

T2DM, a disease of carbohydrate metabolism, should be considered a vascular disease because diabetic patients have higher incidence of atherosclerosis [29]. Metabolic and hematologic abnormalities exist in T2DM, including hyperglycemia, insulin resistance, dyslipidemia, inflammation, and thrombophilia [30]. Diabetes itself is an independent risk factor for cardiovascular events. T2DM increases the risk for CAD by 2 to 4 times that of the overall population [31]. CAD is more ubiquitous and has a worse prognosis in adults with diabetes mellitus than non-diabetic patients [32]. Restenosis in patients undergoing PCI results in a lowered long term survival rate and increased rates of repeat revascularization [33]. Moreover, the likelihood of target vessel revascularization in patients with diabetes mellitus after PCI is increased [34]. A serial intravascular ultrasound study showed that neointimal formation was closely related to diabetes mellitus restenosis after vascular injury [35].

The tagging of newly synthesized DNA in cells or tissues to identify proliferation is an important experimental technique. ^3^H]Thymidine has been quite useful for studying DNA replication and assessing cell proliferation. However, the procedure is burdensome. BrdU staining was believed to be the “gold standard” method for detecting DNA synthesis. However, detection of BrdU requires DNA denaturation of the specimen which makes the staining process difficult and penetration of BrdU-antibody through fixed tissue is comparatively difficult. Recently, the use of EdU has been described to detect DNA replication *in vivo* and *in vitro* in animals. The application of EdU was also reported in plants and fission yeast [36,37]. Grenier *et al.* determined the impact of paternal exposure to cyclophosphamide, an anticancer alkylating agent, on the formation, chromatin origin, and function of micronuclei in cleavage stage rat embryos using EdU incorporation to monitor DNA synthesis [38]. In addition, Škalamera *et al.* transferred protein-coding human open reading frames (ORFs) from the Mammalian Gene Collection into lentiviral expression vector using the highly efficient Gateway recombination cloning and labeled transduced cells with EdU to detect cells progressing through S phase [39]. The full potential of EdU in biomedical research remains to be explored.

Carotid artery injury was induced by balloon de-endothelialization in our previous study. Cell proliferation in obese Zucker rats was higher than in lean Zucker rats at day 7 after injury, and the neointimal area of obese Zucker rats was also broader than that of lean Zucker rats at day 7 after injury [26]. Since Zucker rats and GK rats performed the same in this research. GK rats were used for some of the following research to replace Zucker rats. Time course of neointimal formation in our study was in agreement with experiments performed in our previous study [26]. We have evaluated the effect of rosiglitazone on VSMCs proliferation in Zucker obese and lean rats after carotid artery injury with the use of BrdU incorporation to assess DNA synthesis *in vivo*. The rats received intraperitoneal injections of 50 mg/kg BrdU at hours 18, 12 and 2 before euthanasia. Following this procedure, the number of BrdU-labeled positive cells in the intima and media was counted [23]. In the present day, GK and Wistar rats on the 7th day after injury were chosen to detect EdU-labeled DNA synthesis of neointimal formation and for making morphological comparisons. In the current study, we report the use of EdU to conveniently and quickly detect DNA synthesis of neointimal formation in rats after injury. EdU was injected into model rats at different doses and different injection frequencies to reach an optimal injection method for staining. Better staining results were obtained with multiple EdU injections of 100 mg/kg. This may be related to the fact that more EdU can be incorporated into proliferating cells during DNA synthesis of neointimal formation than a single injection or low doses. Attempts to obtain higher fluorescence intensity and more positive cells by injecting EdU at 200 mg/kg were unsuccessful. Along with enhancement in fluorescence intensity of EdU positive cells, the brightness of the background was simultaneously increased; however, the actual percentage of EdU positive cells remained almost the same.

PI3K/Akt signaling plays a key role in essential cellular functions such as cell growth and survival [40,41]. Aberrant regulation of the PI3K/Akt pathway has been discovered in insulin resistant T2DM, leading to enhanced Akt activity [42]. Activation of Akt increases translation of cell cycle-associated genes, such as cyclins and cyclin-dependent kinases. The entry of vascular cells into cell cycle plays an important role in the pathogenesis of post-angioplasty restenosis [43]. Immediately after injury, VSMCs leave their resting state and enter the cell cycle. Arterial injury results in the proliferation and migration of VSMCs into the intimal layer of the arterial wall. The contribution of vascular proliferation to the postangioplasty restenosis is particularly important. In addition, the proliferative response is also reflected by PCNA activation, which is a well-accepted marker of cell proliferation and assists in DNA replication. In a study of rat aortic VMSCs, Akt signaling is highly activated following stimulation with platelet-derived growth factor (PDGF) [44]. Therefore, we established an *in vivo* rat carotid artery injury model and determined whether neointimal SMCs exhibit activated Akt signaling. Cell proliferation involves changes at the levels of gene transcription and protein translation. In our study, cell proliferation was assessed by using immunohistochemistry staining for PCNA expression and localization to identify the actively cycling cells within the media and intima. The results showed that there were significantly more PCNA-positive VSMCs in diabetic rats than in non-diabetic rats. Using Western blot analysis we found that the protein levels of PCNA and p-Akt were both significantly increased in injured arteries, and were much higher in diabetic rats. qRT-PCR detection of PCNA mRNA levels revealed similar results, which was consistent with the detection of EdU-labeled DNA synthesis. Therefore, we indirectly and strongly confirmed the accuracy of the EdU incorporation and EdU staining by detecting PCNA expression with three distinct molecular biological methods.

To summarize, carotid artery balloon catheter injury led to cell proliferation, which was validated at the levels of PCNA and p-Akt protein as well as PCNA mRNA. And we concluded that EdU incorporation and staining was useful means for detecting DNA synthesis in the vascular neointima quickly and efficiently. Additionally, it only takes less than 2.5 hours for EdU staining, compared to 5 hours and overnight incubation for BrdU antibody detection method. In this work, we also compared the percentages of EdU-positive cells in GK and Wistar rats. We discovered that at day 7 after catheter balloon injury, DNA synthesis in the vascular neointima could be more readily observed in GK rats than in Wistar rats by intraperitoneal injections of EdU at a dose of 100 mg/kg three times. Therefore, it is recommended that a saturated dose and multiple injections can be used to obtain reliable and accurate results. In conclusion, we demonstrate the suitability of EdU incorporation and staining in GK and Wistar rats, and further probe the pathological features of diabetes mellitus by observing a higher amount of DNA synthesis in GK rats.

## Competing interests

The authors declare that they have no competing interests.

## Authors’ contributions

JG contributed to the *in vivo* experiments, participated in the pathological evaluation, interpretation, and acquisition of data, performed statistical analysis, drafted portions of the manuscript, and critically revised the manuscript before final approval. DL participated in the overall design, analysis and interpretation of the data, as well as the writing and presentation of this work. SB participated in the animal model experiments, the data collection and interpretation, performed statistical analysis, drafted portions of the manuscript, and critically revised the manuscript before final approval. TX contributed to the establishment of the animal model as well as the data collection and interpretation. ZZ participated in the model design and the overall conduct of the study. YZ contributed to the study design and data interpretation. All authors read and approved the final manuscript.
